# Interaction between Dietary Fibre and Bioactive Compounds in Plant By-Products: Impact on Bioaccessibility and Bioavailability

**DOI:** 10.3390/antiox12040976

**Published:** 2023-04-21

**Authors:** Vanesa Núñez-Gómez, Rocío González-Barrio, María Jesús Periago

**Affiliations:** Department of Food Technology, Food Science and Nutrition, Faculty of Veterinary Sciences, Regional Campus of International Excellence “Campus Mare Nostrum”, Biomedical Research Institute of Murcia (IMIB-Arrixaca-UMU), University of Murcia, 30100 Murcia, Spain

**Keywords:** by-product, dietary fibre, antioxidant, valorisation, circular economy, (poly)phenols, carotenoids, glucosinolates

## Abstract

In Europe, around 31 million tonnes of food by-products are generated during primary production and trade. The management of these by-products may cause a negative impact, both at the economic and environmental levels, for both industry and society. In this regard, taking into consideration that these by-products retain the dietary fibre compositions and the bioactive compounds of the starting materials, plant food agro-industries have an interest in taking advantage of them, from a nutritional point of view. Therefore, this review evaluates the role of dietary fibre and bioactive compounds in these by-products as well as the potential interactions of both components and their implications for health, since the bioactive compounds associated with fibre may reach the colon, where they can be metabolised into postbiotic compounds, providing important health benefits (prebiotic, antioxidant, anti-inflammatory, etc.). Consequently, this aspect, on which there are few studies, is very relevant and must be considered in the revaluation of by-products to obtain new ingredients for food processing with improved nutritional and technological properties.

## 1. Introduction

Fruits and vegetables play an important role in the diet. Recognition of this, coupled with increasing health concerns and global population growth, has led to an increase in the production of fruits and vegetables [1]. Consequently, the generation of by-products has increased. The Food and Agriculture Organization of the United Nations (FAO) estimated in 2011 that at least one-third of food production is lost or wasted throughout the food supply chain (around 1.3 billion tonnes per year). The highest values were observed for horticultural products, reaching up to 60% [2]. Both food loss and waste cause economic and environmental problems for the food industries because of the large amounts of wasted resources [3]. Therefore, it is necessary to implement research and development actions in order to valorise these materials.

By-products generated within this sector include pulp, peel, seeds, skin, pomace, husks, pods, and stems, all of which contain significant amounts of dietary fibre and bioactive compounds ((poly)phenols, carotenoids, glucosinolates, etc.) in their raw materials [4]. For this reason, the valorisation of by-products can be attractive for the food industry, in order to obtain ingredients of nutritional interest [5].

This, together with the growing concern of policymakers to promote the transformation of the food system through a sustainable production model and the adoption of more sustainable lifestyles by consumers, has led researchers to search for new applications for these by-products. The studies have focused on the implementation of circular economy models in which by-products are reintroduced into production chains as new products [6]. Several studies have been carried out to obtain dietary fibres from these by-products, while others have focused on the extraction of bioactive compounds [7,8], mainly (poly)phenol compounds. However, few studies have focused on the interaction between dietary fibre and bioactive compounds which may be found in the new products obtained after valorisation. Consequently, this review highlights the importance of the utilisation of fruit and vegetable by-products to obtain extracts or potential ingredients with nutritional added value based on their contents of dietary fibre and bioactive compounds, taking into consideration their interaction and bioavailability, as well as their implications for human health.

## 2. Importance of By-Products for the Agri-Food Industry

### 2.1. Generation of By-Products and Their Implications

According to Directive 2018/851 of the European Union (EU), a by-product is a substance or object resulting from a production process whose primary purpose is not the production of that substance or object, and which is not considered as waste, for which different conditions must be fulfilled (Table 1) [9].

**Figure 1:**
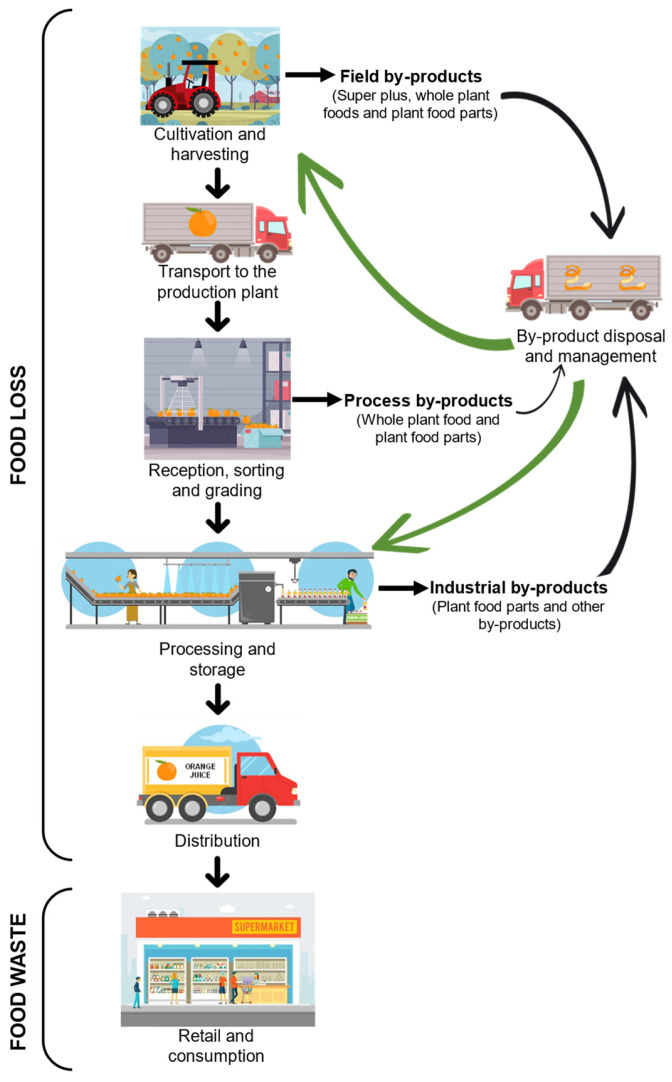
General diagram of by-product generation and management along the food chain. The diagram is based on information that is included in references [2,10].

Related to the activity of the plant food industries, several by-products are generated which could be considered as raw materials for other industrial processes. The most practical classification is made in terms of the moment at which they are generated within the agri-food chain (Figure 1) [10,13]. In this respect, field by-products are those that remain after harvesting, including leaves, stems, roots, and seeds—and even some of the edible parts of plants—that are considered surplus and are not harvested. Subsequently, plant foods are subjected to sorting, grading, and/or cleaning processes, according to specific quality criteria, leading to the generation of process by-products, including husks, straw, leaves, stubble, and shells, among others, which are separated during processing. Whole fruits and vegetables removed because they do not meet quality standards are usually produced as field or process by-products, depending on whether they are discarded prior to processing or during processing (Figure 1). Finally, industrial by-products are those generated from different industrial transformation processes that are by-products of varying physical and chemical materials, including peels, pulp, pomaces, and cakes, among others (Figure 1 and Table 1).

In general, food by-products can be classified as food losses when they are generated at the production, postharvest, and processing stages in the food supply chain. When food losses occur at the end of the food chain (retail and consumption), they are classified as food waste [2] (Table 1). Worldwide, 75% of total food by-products are the result of food loss occurring during production and postharvest, and the total per capita value for both food loss and waste is estimated at 1532 kg/year [2]. The greatest losses around the world for fruits and vegetables occurred in the preharvest and distribution steps, according to the 2016–2017 food loss and waste database, which highlights the importance of taking advantage of the by-products generated at these stages [14]. In the EU, 88 million tonnes of food are wasted every year, equal to a cost of EUR 143 billion, of which 35% are lost between primary production and trade [15]. In plant food industrial processing, the main by-products produced are peels (3–40% of the total fruit and vegetable fresh weight), seeds (15–40% of the total fruit fresh weight), pomaces, and cores, among others [16].

Regarding environmental problems, global food loss and waste are estimated to generate a total carbon footprint of 3.6 gigatonnes of CO_2_ eq. Due to the problems that by-products are generating and will continue to generate in the coming years, valorisation of by-products is included in the Sustainable Development Goal 12 “responsible consumption and production”, since this goal could be achieved by reducing food loss and waste, decreasing the impact on climate change [17]. Moreover, in most cases, by-products are made up of large amounts of biodegradable compounds that can be used by bacteria and, consequently, are susceptible to content pathogens that may cause communicable diseases [18].

From an economic perspective, a reduction in food loss and waste and the valorisation due to their reintroduction into the food chain may lead to a significant reduction in by-product management costs. At the same time, industries may profit from the products that have been obtained from these by-products [18]. In other words, increased profitability along the food chain is achieved through the efficient use of materials. This consists of using materials that would otherwise have been discarded and doing so in an efficient way (e.g., reusing by-products as raw materials, recovering solvents, and reusing water in different processes). In this way, the costs of purchasing new raw materials are saved and the valorisation of a by-product is gained. In addition, through an industrial process, value has been added to the by-product by taking advantage of its properties [19].

In this regard, two new concepts have been introduced in recent decades: the circular economy and the bioeconomy. These are interlinked and give rise to a new concept, “the circular bioeconomy”, which is focused on the sustainable production of renewable resources (Table 1) [12].

### 2.2. Composition of Agro-Industrial By-Products

A strategy for the utilisation of by-products from the agri-food industry should take into consideration their composition. In this regard, these by-products can be a good source of proteins, dietary fibre (DF), starch, micronutrients [20], as well as lipids. Seeds are the by-products with the highest contents of fat. For example, fat constitutes more than 20% of pepper and melon seeds [21,22]. However, the content of DF stands out in some plant by-products, as described in the scientific literature for some by-products; this is shown in Table 2. The contents of total dietary fibre (TDF) in apple, redcurrant, rowanberry, and tomato pomace are 51.1%, 58.1%, 67.2%, and 64.1%, respectively (Table 2) [23,24]; whereas in citrus, mango, plantain, and tomato peels, the mean contents of TDF are 67.4%, 69.9%, 64.3%, and 86.2% (Table 2), respectively [25,26,27], while in broccoli stalks it is around 38%. On the other hand, the content of TDF in seeds is lower compared with pomace and peels, ranging from 2.9 to 26.3%, while in undifferentiated by-products it is between 35 and 90% (Table 2).

Insoluble dietary fibre (IDF) has been described as the major component of TDF in these by-products, being represented by cellulose, hemicellulose, and lignin [28]. The amounts of soluble dietary fibre (SDF) range from 3 to 25%, represented mainly by pectin contents, with a high percentage of TDF in mango peel and passion fruit by-products [28]. In addition, plant food by-products are rich in antioxidant bioactive compounds ((poly)phenols, carotenoids, and glucosinolates), demonstrating different compositions according to the raw materials (Table 2). Other authors have reported that grape, tomato pomace, and red corn cob contain significant concentrations of gallic acid, rutin, epicatechin, and apigenin [25,33]. Relevant contents of carotenoids have also been reported in orange, mango, carrot, and tomato by-products [25,34], while glucosinolates have been reported in broccoli stalks [30].

### 2.3. Industrial Applications

In the agro-industrial sector, about 50% of the mass of fruit and vegetable products are discarded during the course of the industrial process [35]. Traditionally, landfilling or incineration has been used for by-product disposal, but these practices lead to the problem of environmental pollution (air, water, and soil). Although they are now in disuse, these practices are still actively used in some countries [18]. As an alternative, these by-products have been also used as fertilisers in agronomical practices and/or as animal feed, since they still maintain the chemical compositions of the raw materials and provide various amounts of protein, cellulose, and hemicellulose [19]. However, the utilisation of by-products in animal nutrition poses certain problems, such as the presence of toxic or chemical compounds which could be dangerous for the livestock, affecting performance and food production [36].

Despite the application of plant foods, by-products have been used for many years. Nowadays, new trends in by-product utilisation have focused on the recovery and reuse of valuable components. This aspect has attracted increased interest from companies, mainly due to the application of circular economy and bioeconomy approaches. In this regard, by-products can be used to obtain compost, biofuel, bio-adsorbent materials, bio-polymers, and new materials for textiles [37].

Other recent applications are the extraction of different bioactive compounds for the food industry and nutraceutical and pharmacological applications [18], since by-products from fruits and vegetables are rich in phytochemicals, which are considered natural active substances with significant health-promoting effects [38]. Once extracted, the phytochemicals or the bioactive compounds can be used as nutraceuticals, as in the case of extracts from berry pomace [39]. In addition, other by-products have shown pharmacological properties, as in the case of orange peel components, which showed potential use in the prevention of membrane oxidative stress [40], and berry pomace, with potential use in antimicrobial therapies [41]. Orange juice by-products have shown neuroprotective properties [42], and flavonoids from citrus peel have shown potential for cancer prevention [43].

Furthermore, new uses have also been reported within the cosmetics industry. In this regard, grape by-products could be used to treat skin wrinkling and pigmentation disorders [44]. Other in vitro and in vivo studies have linked (poly)phenols to the prevention of skin damage, rosacea, and psoriasis, among other disorders [45]. Compounds such as β-carotene and lycopene (from tomato peels) have been used in the development of sunscreen formulations [45].

Nowadays, due to the chemical properties and compounds present in fruit and vegetable by-products, the reintroduction of these by-products into the food chain is one of the most important paths to recovery, as they can be reused in the food chain following the circular economy model. For example, carotenoids and (poly)phenols obtained from plant by-products have been used as natural food colorants and preservatives [46]. Moreover, betalains have been used as natural colorants [47]. The natural colorants extracted from industrial by-products can be considered new ingredients and allow the development of innovative foods with natural or sometimes fortified ingredients, with artificial colorants being replaced by ones with higher nutritional values and which are often cheaper [48]. Another important use of plant food by-products is the extraction of TDF, IDF, and SDF, which can be used as ingredients for the design and development of fibre-rich products, which are important for maintaining digestive health and preventing chronic diseases. [49]. Fibre, such as that from fruits, vegetables, and grains, can also act as a natural thickener, emulsifier, and stabiliser in food products [50]. In view of this, several food fortification studies have been carried out with fibre-rich ingredients. In these studies, for example, fruit and vegetable fibre powders were added to bakery products, such as bread and cakes, to increase their fibre content and improve their texture. More specifically, apple pomace and cactus powder were added to improve the technological properties of cakes, and mango skin powder was added to soft biscuits. In meat products, such as sausages and burgers, fibre by-products can be used as binders to improve the texture and reduce the fat content, as in the case of grape fibre, which has been used in the manufacture of chicken patties and minced fish muscle [51]. Moreover, oat fibre and psyllium husks are used in breakfast cereals and snacks to increase their fibre content, promote satiety, and prevent cholesterol accumulation; in dairy products, such as yogurt and ice cream, they can be used to improve the creaminess and prevent the formation of ice crystals, and in sauces and dressings they can be used as thickeners and stabilisers to improve texture and prevent separation. Finally, other recent applications are being developed to improve the technological and nutritional properties of vegan or gluten-free recipes, as in the case of chia fibres that are used as egg substitutes, acting as binding agents. Therefore, the use of vegetable fibre by-products in the food industry offers a range of benefits, both in terms of improving the nutritional value of food products and enhancing their functionality [52,53,54,55].

## 3. Main By-Product Components

### 3.1. Dietary Fibre

Dietary fibre has been extensively studied because of its physicochemical properties, which are directly related to its physiological effects. However, there is still no consensus on the definition of DF, as it has been changing over time according to different authors. The European Food Safety Authority (EFSA) defines DF “as non-digestible carbohydrates plus lignin and hence includes: non-starch polysaccharides (NSP) (cellulose, hemicelluloses, pectins and hydrocolloids, e.g., gums, mucilages, β-glucans), resistant oligosaccharides (fructo-oligosaccharides, FOS, and galacto-oligosaccharides, GOS), other resistant oligosaccharides, resistant starch (RS) (consisting of physically enclosed starch, some types of raw starch granules, retrograded amylose, chemically and/or physically modified starches), and lignin associated with the DF polysaccharides” [56]. The Food and Drug Administration (FDA) defines it “as non-digestible soluble and insoluble carbohydrates (with 3 or more monomeric units), and lignin that are intrinsic and intact in plants; as well as isolated or synthetic non-digestible carbohydrates (with 3 or more monomeric units), which have physiological effects that are beneficial to human health” [57].

The main compounds of DF are the polysaccharides of the cell wall, which are mainly composed of cellulose, hemicellulose, pectin, and other components, such as gums, starch, oligosaccharides, and lignin [58]. Cellulose is the most abundant biopolymer in nature and is found in high amounts in stems, straw, and peels, constituting 42% of banana stems, 51% of maize straw, and 41% and 39%, respectively, of onion and oat peels [59]. Cellulose has also been found in high proportions in citrus peel, grapefruit wastes, broccoli stalks, and tomato peel fibre (34%, 27%, 19%, and 13%, respectively) [25,30,60]. Hemicellulose is the second most abundant component of lignocellulosic biomass, after cellulose [61,62], and is found in blackcurrant pomace (constituting 25%), chokeberry (34%), cherry (11%), citrus peel (10%), and grapefruit waste (6%) [60,63]. Pectin is mainly found in fruit and vegetable peels, constituting 8–53% of orange peel, 1–17% of lemon peel, and 2–16% of grape skin; these amounts vary according to the different analytical and extraction methods [64].

Moreover, DF can be classified not only according to its chemical compounds but also according to its properties. Each type of fibre shows different properties of solubility, viscosity, hydration, fermentability, and fat absorption capacity, which determine the beneficial effects for human health and the physicochemical applications [65]. In this regard, depending on their solubility, fibres are classified as soluble—showing beneficial effects on serum lipids and glucose metabolism, both directly related to their ability to form gels—or as insoluble fibres, which are those that have a laxative effect on the colon, although it should be noted that this classification is increasingly in disuse [65]. Nowadays, more attention is being paid to other characteristics that determine physiological effects on human health, such as viscosity, which is directly associated with the ability to form gels. Viscosity is more associated with soluble fibres than with insoluble fibres, but not all soluble fibres are viscous. These fibres can bind water and produce the distension effect that is related to satiety and are able to favourably alter biomarkers of cardiovascular disease. Another important characteristic to take into account in determining potential physiological effects is fermentability, which is the ability to be metabolised by colonic microbiota, which alters composition advantageously [65].

### 3.2. (Poly)phenols

(Poly)phenols are compounds derived from the secondary metabolism of plants. They play important roles in reproduction and growth in the plant kingdom and protect plants from adverse environmental factors, pathogens, and herbivores [66]. They are also responsible for some of the organoleptic properties of plants, such as color, taste, and astringency, and their consumption has preventive properties against diseases, such as obesity, diabetes, cardiovascular and neurodegenerative diseases, and some types of cancer [67]. These beneficial properties for human health derive from their antioxidant capacities and from a combination of other factors, such as their interaction with cellular signalling pathways [68].

(Poly)phenols have one or more aromatic or benzyl rings to which one or more hydroxyl groups are attached. Generally, (poly)phenols are not found free, but are linked to sugars by β-glycosidic bonds to a hydroxyl group (O-glycosides) or to a carbon atom of the aromatic ring (C-glycosides). (Poly)phenol content and composition may vary depending on physiological, genetic, and agronomic factors, such as cultivar, soil composition, agronomic treatments, climatic conditions, and pre- and postharvest treatments [69]. More than 8000 compounds have been identified in nature [70]; these are classified according to their origin, structure, and biological function into two main groups: flavonoids and non-flavonoids [71].

Flavonoids are the largest and most studied group of (poly)phenols, with more than 4000 types of compounds identified. Their structures are formed by two aromatic rings joined by three carbon atoms forming an oxygenated heterocycle [72]. Depending on the degree of hydroxylation, oxidation, and saturation of the central pyran ring, flavonoids are divided into several subgroups: flavan-3-ols, flavones, flavonols, flavanones, isoflavones, and anthocyanidins. They occur naturally as glycosides rather than aglycones, mainly bound to glucose, xylose, rhamnose, or galactose [71].

Non-flavonoids are the second largest group of (poly)phenols. They have a simpler chemical structure than flavonoids and include phenolic acids, hydrolysable tannins, stilbenes, coumarins, and lignans [71].

In addition to their chemical classification, (poly)phenols can also be classified according to their binding to food matrix molecules in extractable and non-extractable compounds. Extractable (poly)phenols (EPPs) are low-molecular-weight (poly)phenols that are easily released from the food matrix. EPPs are linked to food components by different weak interactions, such as van der Waals and hydrophobic interactions, hydrogen bonds, and electrostatic forces, and their interactions are broken up during extraction with organic solvents [73,74]. This group includes several classes of compounds, such as flavonoids, phenolic acids, stilbenes, lignans, and hydrolysable tannins, among others [75,76].

On the other hand, non-extractable (poly)phenols (NEPPs) are those which cannot be extracted easily with organic solvents, either because they are macromolecules, because they are bounded to the food matrix and mainly to the cell walls, or because they are high--molecular-weight compounds [76]. NEPPs, once they are synthetised in the cell, are transported into the cell wall, and then conjugated with cell wall macromolecules, such as cellulose and protein, through ester and glycosidic bonds, thus contributing to the formation of the cell wall structure [73]. The DF-NEPP complex has implications for human health since, due to their bonds, NEPPs may reach the colon almost intact and, after their release from DF, if it occurs, the compounds can be used by the bacteria that form the microbiota [77]. Within this group there are two subgroups: hydrolysable (poly)phenols, which are low-molecular-weight (poly)phenols bound to the food matrix—generally to the fibre fraction [78]—and non-extractable proanthocyanidins, which are high-molecular-weight proanthocyanidins with long chains that, due to their structure, limit their release from the food matrix [79]. Notably, more than half of the (poly)phenols of vegetables such as broccoli, carrot, orange, and tomato, are NEPPs [80].

Moreover, vegetable by-products, which are a source of DF, are also a promising source of NEPP compounds [78]. These compounds, due to their distribution in the food matrix, have important beneficial effects on human health due to their antioxidant effects, microbiota modulation, and the biological activities of metabolites produced during NEPP catabolism [80].

The presence of EPPs has been reported in many plant by-products. Chlorogenic acid, among other EPPs, has been reported mainly in coffee pulp and husks, and has also been reported in apple peel and seeds. Other compounds, such as catechin and epicatechin, have been reported mainly in grape by-products [81]. Flavanones and flavones are mainly found in orange by-products, and more specific ones, such as punicalagin and punicalin, are found in pomegranate by-products [81].

### 3.3. Carotenoids

Carotenoids are C_40_ tetraterpenoid pigments widely distributed in plants, algae, fungi, and bacteria. They consist of eight isoprenoid units inverted in the centre of the molecule by a double-molecule system. They can be found either in free form or esterified with fatty acids; the latter type of bonding allows the storage of carotenoids [82].

Carotenoids are located in different parts of plants, such as leaves, roots, fruits, and seeds, and mainly in fatty tissues, as they are fat-soluble compounds. In cells, they are located in photosynthetic tissues, known as thylakoids, together with chlorophylls, some of them helping in photosynthesis by capturing light energy [83].

Carotenoids are classified into two subgroups, according to functional group. Xanthophylls, including lutein, zeaxanthin, and cryptoxanthin, are oxygenated derivatives, as they have oxygen in their structures. The other group is known as carotenes, which are hydrocarbon derivatives and do not have any group attached to their structure; within this group are α-carotene, β-carotene, and lycopene [82].

Carotenoids cannot be synthesised by the human body, so they must be obtained from the diet. Generally, the beneficial effects attributed to these types of compounds are due to their activity as provitamin A, for which they must have an unsubstituted ring structure with an 11-carbon polyene chain. Carotenoids are strong antioxidant compounds, and they are involved in reducing the effects of ageing, which is related to the progressive loss of cellular functions [84]. Specifically, effects have been demonstrated against skin, eye, and vascular ageing, mainly due to the protection against cellular oxidation, due to their ability to scavenge free radicals [84]. In addition, they have beneficial effects in neurodegenerative diseases, such as Alzheimer’s, improving cognitive functions [85]. They also exert a preventive effect against other diseases related to oxidative stress, such as osteoporosis [84]. Furthermore, they reduce the risk of some types of cancer, including breast, prostate, liver, and lung cancer, among others. This anticarcinogenic activity is related to their various mechanisms, since they can act as antioxidants, and, in some circumstances (high oxygen tension and high carotenoid concentration), they may act as pro-oxidants with the opposite effect [84]. Carotenoids have also been reported to have a beneficial effect on cardiovascular diseases through their protective effect against oxidation of low-density lipoprotein (LDL) cholesterol and by reducing blood cholesterol by inhibiting the enzymatic activity of 3-hydroxy-3-methylglutaryl-CoA reductase, which is involved in the cholesterol biosynthesis pathway [86]. They may also produce a hepatoprotective effect, due to their ability to reduce oxidative stress and regulate hepatocyte lipid metabolism, reducing the risk of diseases such as non-alcoholic fatty liver disease [87].

Regarding the contents of carotenoids in plant food by-products, it is recognised that broader series of non-phenolic compounds can be also found in rich dietary fibre extracts [73]. For example, β-carotene, which is an orange pigment, has been described in sweet potato and carrot by-products and in tomato peel and seeds. Lycopene, a red pigment, has also been found in high amounts in tomato by-products [88]. In addition to β-carotene, α-carotene, which is a yellow pigment, has also been found in carrot by-products [89]. β-cryptoxanthin and violaxanthin have been found in orange and mandarin decoction by-products in high amounts [90].

### 3.4. Glucosinolates

Glucosinolates, with more than 120 different chemical compounds identified, are a large group of bioactive compounds. Their structure is based on an *S*-β-D-glucopyrano unit anomerically connected to an *O*-sulfated (*Z*)-thiohydroximate function. The rest of the molecule is known as an aglycone and constitutes the side chain (“R-group”), which has a highly variable structure, depending on the precursor amino acid [91]. Glucosinolates are hydrolysed by a plant enzyme named myrosinase (EC: 3.2.1.147), which hydrolyses the thioglycoside bond to yield metabolites, such as isothiocyanates, thiocyanates, nitriles, epithionitriles, and oxazolidine-2-thione, whose production depends on the content, the kind of the glucosinolate, and the environmental conditions [92]. The contents and compositions of glucosinolates vary depending on several factors, such as genotypic variability, development stage at harvest, environmental and seasonal variations, agricultural practices (irrigation, fertilisation, and elicitation) and other postharvest factors (storage, processing, and packaging) [93]. 

Glucosinolates can be classified according to several criteria, but one of the most common classifications is according to their precursors. Aliphatic glucosinolates include methionine, isoleucine, leucine, alanine, and valine derivatives and are the most common glucoraphanins. Indolic glucosinolates include tryptophan derivatives, most commonly glucobrassicin, whereas aromatic glucosinolates are derivatives of phenylalanine and tyrosine [94].

Glucosinolates are abundant in plant foods of the *Brassicaceae* family, which includes vegetables such as broccoli, brussels sprouts, cabbage, mustard, radish, watercress, and wasabi, among others. In addition to the *Brassicaceae* family, other edible dicotyledons, such as capers, papaya, and moringa, contain glucosinolates [95]. Different glucosinolates have been identified in *Brassicaceae* by-products, such as broccoli stalks, mustard leaves, and winter rapeseed leaves and stems [30,96,97].

Although glucosinolates might act in plants as a reserve of sulphur and nitrogen, their main role is as a defence mechanism against pests, mainly against herbivores. When tissue rupture occurs due to pest damage, myrosinase, which is stored in the idioblasts, is released. Then, it hydrolyses the glucosinolates, producing toxic products, such as isothiocyanates, that act against pests [98]. However, it should be noted that glucosinolates not only respond to biotic stress, but also to environmental stress, since when atmospheric or soil conditions are unfavourable (low temperatures and water deficit) there is an increase in these compounds [99].

Glucosinolates and their metabolites have many beneficial effects on human health derived from their consumption. In this regard, their anticarcinogenic activity stands out, which derives from different mechanisms, including detoxification, as isothiocyanates may alter the metabolic pathways, reducing the activation of procarcinogens and improving their excretion. Other mechanisms include cell apoptosis, ROS-mediated oxidative stress, inhibition of cell cycle progression, angiogenesis, histone deacetylase, or altering oestrogen metabolism [100]. Furthermore, glucosinolates also exhibit an antioxidant effect, but not directly, because they do not scavenge free radicals. However, they are able to remove free radicals by modulating the enzymatic activities involved in the metabolism of xenobiotics, resulting in long-lasting antioxidant activity [101]. In addition, isothiocyanates demonstrate immunomodulatory and anti-inflammatory activities, which are directly related to several chronic diseases [102]. The consumption of foods rich in glucosinolates has also been linked to a reduction in endogenous cholesterol synthesis, thereby helping to prevent coronary heart disease [100]. Moreover, their properties have also shown a beneficial effect against neurological diseases and diabetes [103].

The information in the literature regarding whether glucosinolates can be found bound to fibre or not is inconclusive. In this regard, in a previous study that we performed with broccoli stalks, we only found extractable glucosinolates, agreeing with the results observed by other authors [30,104]. However, glucosinolates are found in the sections of fibre obtained after extraction, as glucosinolates remain trapped in the vegetable structures, with glucoraphanin being the main one identified in broccoli by-products [105].

### 3.5. Antioxidant Dietary Fibre

As DF and (poly)phenols are found in significant concentrations in fruits and vegetables, the presence of bioactive compounds bound to the components of DF should be considered from a chemical and nutritional point of view. These bioactive compounds are responsible for the antioxidant activity of fibre, which in turn determines the beneficial effects of DF on health [106]. In this regard, the antioxidant activities of different by-products analysed by different methods are included in Table 3, where avocado seed and orange peel stand out.

In 1998, Saura-Calixto (1998) defined the concept of antioxidant DF, which refers to plant food material that contains at least 50% of total DF on a dry matter basis, with the following requirements, according to its antioxidant capacity: 1 g of the material must have the capacity to inhibit lipid oxidation equivalent to 200 mg of vitamin E and to eliminate free radicals equivalent to 50 mg of vitamin E. These capacities must be intrinsic and not derived from the addition of antioxidants. Hence, the properties of antioxidant DF are related to the bioactive compounds that are attached to the components of the cell walls, and the intrinsic properties depend on the chemical characteristics of these compounds. Therefore, antioxidant DF is a component that contains both a high proportion of DF and significant amounts of natural antioxidants associated with the fibre matrix [107].

The main antioxidant compounds of DF are (poly)phenols, previously described as NEPPs. (Poly)phenols are found enclosed by the tonoplast and cytoplasmic lipid membranes in the vacuoles of plant cells [108]. The aromatic rings of these compounds have hydrophilic groups that can bind to polysaccharides or cell wall proteins. Regarding interaction with proteins, there are two types of potential links: covalent and non-covalent interactions. The most common links are the non-covalent ones; these are reversible and influenced by amino acid sequence, conformation, and other conditions, such as temperature, pH, and ionic strength. The most frequent interactions between proteins and (poly)phenols are hydrophobic and/or hydrogen bonds [108]. Regarding the binding that takes place in the carbohydrate and (poly)phenol interactions, they occur between the (poly)phenol hydroxyl group and the oxygen atom from the glycosidic bond of the polysaccharide [109]. The most common bonds are non-covalent bonds of weak energy, mainly hydrogen bonds and hydrophobic interactions, which increase with the degree of polymerisation [109]. This effect is very similar to that occurring with proteins, but with a clear difference, as the interaction with cell walls is fast and immediate, and no aggregation or precipitation occurs in the binding with simple (poly)phenols or with procyanidins—an effect that occurs with proteins [109]. Covalent interactions between cell walls are rare; they occur in some cases, such as in the formation of pomace during juice extraction, in which case the covalent bond may be mediated by the oxidation of procyanidins during enzymatic browning [109]. This interaction is determined by factors such as the porosity of the microstructure, as the pore size restricts the entry of certain (poly)phenol molecules, and other factors related to (poly)phenol characteristics, the most important being molecular weight [110].

DF is assumed to be composed of different (poly)phenols present in plant foods. Phenolic acids, including ferulic acid, sinapic acid, and *p*-coumaric acid, among others, are mainly found in cereal fibres [111]. On the other hand, flavonoids have been described as the major (poly)phenols in citrus fibre [112], and hydrolysable tannins have been found mainly in pomegranate and nut fibres [113,114].

**Table 3:** Antioxidant activities (µmol TE/g) of different by-products analysed by different methods.

Fruit/Vegetable	FRAP	DPPH	ABTS	ORAC	Reference
Avocado peel	-	52–190	-	58–631	[115]
Avocado seed	-	128–240	-	229–464	[115]
Papaya peel fibre	25	0.6	-	-	[116]
Orange peel	75–155	134–269	489–810	-	[40]
Broccoli stalk fibres	1–2	-	-	17–19	[30]
Tomato peel fibre	-	-	3.9	-	[25]
Raspberry fibres	2–38	-	-	31–81	[117]
Chokeberry pomace	-	3	8	-	[118]
Persimmon flour	22	13	10	-	[119]

Trolox equivalents (TEs); ferric reducing antioxidant power (FRAP); 1-1-diphenyl-2-picrylhydrazyl (DPPH); 2,2′-azino-bis(3-ethylbenzothiazoline-6-sulfonic acid) (ABTS); oxygen radical absorbance capacity (ORAC).

Nevertheless, it should be noted that the antioxidant capacity of DF is not only due to the presence of (poly)phenols, but also, although in smaller quantities, to a rich fibre component. Other compounds, such as carotenoids, melanoidins, or Maillard reaction products, may also provide these properties [110].

The presence of these compounds in by-products has important implications for human health, as they could have beneficial effects, such as the prevention of constipation and the promotion of laxation, reduction in postprandial blood glucose, and the lowering of insulin response and cholesterol levels [120]. One of the most documented effects for DF and (poly)phenols is the prebiotic effect. After microbiota metabolisation of these components, other compounds, such as short-chain fatty acids (SCFAs), are generated [65]. These SCFAs act as energy substrates for colonic epithelial cells and decrease the pH of the colon environment, reducing, at the same time, the presence of pathogenic bacteria, helping to prevent certain diseases [121,122].

## 4. Bioaccessibility and Bioavailability of Bioactive Compounds

### 4.1. (Poly)phenol Bioavailability

To study the bioaccessibility and bioavailability of bioactive compounds present in foods, several factors must be taken into account, including the matrix, food composition, and processing, since the intermolecular bonds that may occur, as well as the molecular structure and size, may affect the digestion and the availability of the compound to be absorbed in the gut [123]. It has been reported that these factors affect phenolic compounds’ bioaccessibility and bioavailability [124], but other host-related factors, such as gender, age, pathologies, genetics, physiological condition, enzyme activity, and microbiota status, among others, should also be taken into consideration [125]. 

Moreover, (poly)phenol structure affects bioaccessibility and bioavailability, and it has been determined that, depending on class, bioavailability is ranked as follows: phenolic acids > isoflavones > flavonols > catechins > flavanones, proanthocyanidins > anthocyanins [126].

**Figure 2:**
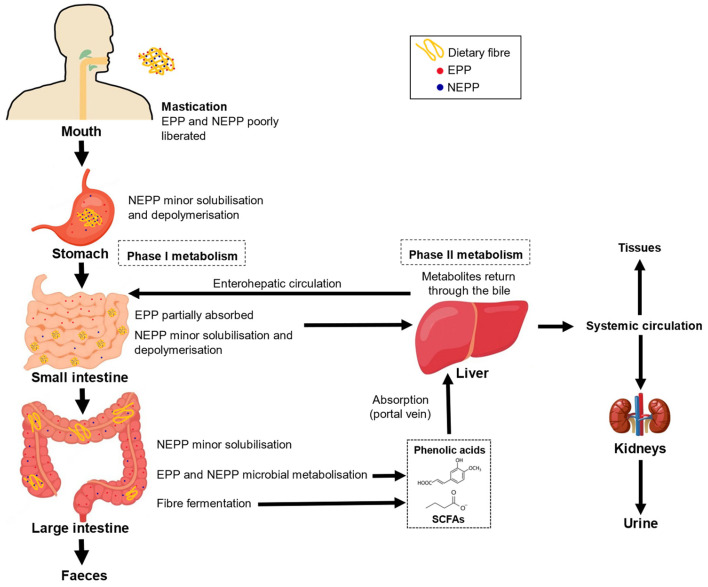
Metabolic fate of extractable (poly)phenols (EPPs) and non-extractable (poly)phenols (NEPPs). The digestion process of dietary fibre and polyphenols linked to dietary fibre is based on the information from Martínez-Meza et al. (2021) [80].

According to the classification of (poly)phenols in EPPs and NEPPs, as is shown in Figure 2, EPPs may be absorbed partially in the intestinal enterocytes of the upper part of the small intestine, because they are not bound to other food components and are, therefore, bioaccessible. The EPPs that are not absorbed in phase I metabolism pass into the colon, where they undergo conversion by microbiota. After microbiota biotransformation into catabolites, some of them may exert gut benefits or may be absorbed in the colon and transported through the portal vein to the liver, where phase II metabolism takes place (Figure 2). Phase II metabolites enter the bloodstream and are finally excreted in urine, while those that are not absorbed are excreted in faeces [80].

On the other hand, NEPPs need to be unbounded from molecular interactions with food components, such as fibres, proteins, or lipids; hence, they must be bioaccessible before being absorbed (Figure 2). Digestion starts with a mechanical separation by mastication and oral grinding; saliva also helps to release (poly)phenols, as it contains enzymes with β-glucosidase activity. This release continues in the stomach and small intestine through the release of gastric acid and enzymes that hydrolyse the macrostructures of the food components [127,128]. Finally, (poly)phenols that have been released during the digestion process and are soluble can diffuse for absorption in enterocytes. They may reach the bloodstream after several metabolic processes, but it should be noted that the solubilisation and consequent absorption of NEPPs in this digestion stage is minimal [80]. Most NEPPs can reach the lower gastrointestinal tract almost intact due to their binding to other food components [77], mainly DF. When NEPPs reach the colon, depending on the extent to which DF is fermented, they can be released from the food matrix and then used by the intestinal microbiota and transformed into catabolite products (Figure 2). These catabolites follow the same process as those produced by EPPs after microbial metabolisation (phase II metabolism) [80].

Health benefits derived from (poly)phenol consumption are related to their antioxidant capacity and to a combination of other factors, such as their interaction with cellular signalling pathways [68]. As mentioned in the previous sections, this effect is related to the free radical scavenging capacity, reduction of iron (III) ions, and inhibition of lipid peroxidation [129]. In this regard, most of the current studies only take into account the antioxidant capacities of EPPs, which are extracted with organic solvents from the food matrix, while several studies have shown that NEPPs significantly increase the antioxidant capacity of the food matrix [130,131]. In addition, due to interactions with DF, (poly)phenols have other beneficial effects, which are described below.

The microbiota may use the (poly)phenols by dihydroxylation, ester cleavage, decarboxylation, and ring cleavage, among other metabolic processes [132]. Through these processes, microbiota can metabolise (poly)phenols, increasing the number and profile of microorganisms with several beneficial effects for the host’s health, demonstrating a prebiotic-like effect. In this respect, they promote the growth of *Lactobacillus* and *Bifidobacterium* [133] and have an effect on the positive modulation of *Akkermansia muciniphila* and *Faecalibacterium prausnitzii*, which have been described as antiobesity bacteria [80,134]. They also may increase the *Firmicutes*/*Bacteriodetes* ratio, which is related to beneficial host health effects [135]. Furthermore, these compounds are able to reduce numbers of pathogenic species, which also benefits the growth of beneficial microbiota [132]. It should be noted that (poly)phenols have other mechanisms for modulating the microbiota, such as antimicrobial effects, inhibition of microbial enzymes, and alterations to mucus viscosity [136].

The catabolites derived from the metabolism of EPPs and NEPPs are the same, but NEPP derivatives are absorbed with some delay compared to the ones derived from EPPs, which may cause prolongation of their biological activity over time [80]. Catabolites reported from (poly)phenol metabolism include urolithins, which are derived from ellagitannins and ellagic acid metabolism [137]; phenolic acids (phenylpropionic, phenylacetic acid, and benzoic acid derivatives), derived from flavanones, flavonols, and anthocyanin metabolism [138]; and γ-valerolactones and phenylvaleric acid, derived from flavan-3-ol metabolism [139]. These catabolites exhibited some benefits for host health (anticancer, anti-inflammatory, antiglycative, neuroprotective, and antiatherogenic effects, as well as inhibition of lipid synthesis and insulin-modulating activities) [80].

A synergistic potential effect between (poly)phenols and DF has been observed in several studies, with increased SCFA production observed in the presence of (poly)phenols [140]. However, it should be noted that the mechanism is not clear, and a opposite effect has been observed in other studies [141,142]. Further in vitro and in vivo studies are needed to elucidate the simultaneous effects of both substrates on microbiota, taking into account the preference of gut bacteria for the transformation of DF or (poly)phenols [80].

### 4.2. Bioaccessibility and Bioavailability of Other Bioactive Compounds

Carotenoids are mainly found in plant foods and their bioaccessibility and bioavailability depend on several factors, such as food composition and food matrix. Moreover, processing and the presence of oil in the food matrix are important factors, due to the fat-soluble properties of carotenoids. The bioavailability of β-carotene was found to range between 3.5% and 90% [143]. The cooking process helps the release and absorption of carotenoids, and the presence of oil favours this process due to their fat-soluble character; on the other hand, it should be noted that the application of high temperatures may cause degradation, reducing the bioavailability and isomerisation of carotenoids, which, depending on the carotenoid, may increase or decrease their bioavailability [87,144]. However, the presence of other components, such as protein and fibre, can hinder their release and subsequent absorption [87,145]. 

Therefore, the first step, prior to digestion, performed by mechanical processes carried out during mastication, is critical, as it releases carotenoids from cell structures, since they must be released from the plant cell walls and then from the chromoplasts. After the release of the carotenoids, they must diffuse into a lipid emulsion and then must be solubilised by pancreatic lipases and bile salts, leading to the production of mixed micelles, which finally allow absorption in the small intestine and entry into systemic circulation. Micelle assembly is critical for the bioaccessibility and bioavailability of carotenoids, and thus constitutes a factor affecting their beneficial effects for human health [82].

When carotenoids are not included in micelles, they are able to reach the colon. Micelles can also reach the colon, but to a lesser extent. To our knowledge, scant information has been obtained on how the microbiota can metabolise these compounds and regarding the potential metabolites that are produced. However, it has been observed that they accumulate in the colon, as they cannot be absorbed, which is important for the potential beneficial effects that they may have on microbial communities and colonic cells [146,147]. In this regard, apo-carotenoids have been described as potential metabolites possessing the ability to react with different transcription factors (e.g., Nrf2, RARs, RXRs, and PPARs) and regulate key pathways of lipid metabolism; they may also stimulate the expression of phase II enzymes with antioxidant, cryoprotective, or immunomodulatory activities [144,147]. The capacity of several sporulated bacteria belonging to the *Bacillus* genus to synthesise apo-carotenoids de novo has been observed [148,149]. In another study, it was described that supplementation with *B. indicus* spores improved metabolic syndrome symptoms in rats, although there were no changes in the microbiota, suggesting that the benefits could be mediated by bacterial metabolites [150]. Furthermore, in an in vitro study, it has been reported that fucoxanthin inhibits the growth of *Escherichia coli* and promotes the growth of *Lactobacilli* via metabolism that may involve bacterial metabolisation of fucoxanthin to fucoxanthinol [151].

Glucosinolates may also be present in DF by-products obtained from *Brassicaceae*, due to their occurrence in various plant foods. The bioaccessibility and bioavailability of glucosinolates depends on several factors, including the concentration of glucosinolates and their hydrolytic products in the food, the concentration of myrosinase and its stability, the effects of processing and storage, and, in particular, the physico-chemical characteristics of glucosinolates, the digestive process, and other host characteristics [152]. The bioavailability of different glucosinolates and isothiocyanates was found to range from 0.7 to 80% [153]. After ingestion, a small proportion are absorbed directly in the stomach, and most of them reach the small intestine. There plant myrosinase, when it is presented, can hydrolyse glucosinolates, yielding isothiocyanates as hydrolysis products, which can be absorbed. Due to the fact that plant materials containing glucosinolates are mostly consumed cooked, myrosinase enzymes are inactivated, which may lead them to reach the colon intact [154]. In addition, myrosinase activity has been described in several microbial strains; consequently the metabolism of glucosinolates varies depending on the composition of the microbiota of the consumers. In this regard, the production of erucin, erucin nitrile, iberverin, and iberverin nitrile from the degradation of glucoerucin, glucoiberin, and glucoraphanin by a selection of human gut bacteria has been reported [155]. These glucosinolate derivatives may have an important impact on human health, such as anticancer and anti-inflammatory effects [156].

## 5. Dietary Fibre-Rich Functional Foods and Claims

The production of fibre-rich ingredients may allow their use in the food industry for the development of functional foods from by-products [51]. Thus, functional foods have been defined as “natural or processed foods that contain biologically-active compounds; which, in defined, effective, non-toxic amounts, provide a clinically proven and documented health benefit using specific biomarkers, to promote optimal health and reduce the risk of chronic/viral diseases and manage their symptoms” [157]. For the development of this type of food, fortification is used as a technique. Fortification consists of adding ingredients with functional properties to different foods to improve their biological activities [158,159]. This technique has been widely used in industry for the development of bakery and meat products. Fibre-rich plant extracts and bioactive compounds are the main ingredients used for this purpose. Other foods, such as beverages, are enriched with fibre, with the aim of replacing the fibre content that has been removed during industrial processing, thus reducing the sugar content. Usually, soluble fibres are preferably used for their dispersion capacity, although sometimes other types of fibres are used in products, such as cloudy beverages, or more viscous fibres to stabilise beverages [160]. Soluble fibres are also used in jam production to reduce the caloric content without comprising its sensory properties [161].

## 6. Conclusions

Fruit and vegetable production generates many by-products which, due to their similar composition to the starting materials, can be valorised as new ingredients. The new ingredients can be important sources of fibre and bioactive compounds, mainly (poly)phenols, but also, notably, carotenoids and glucosinolates, according to the characteristics of the raw materials. In many cases, the binding or presence of these compounds, together with fibre, gives the former an antioxidant character and provides important beneficial effects for the health of consumers. These include prebiotic, antioxidant, neuroprotective, anticancer, and cardiovascular-risk-reduction effects, among others. 

Although there are several research studies on the differential metabolism of (poly)phenols bound or not to fibre, it would be of interest to extend research in this field and to study the potential interactions of fibre in the metabolism of carotenoids and glucosinolates at the colonic level, research on which is very scarce. Since the metabolites generated in the metabolism of fibre may directly influence the metabolism of bioactive compounds, the fact that they are bound in the food matrix may be a determining factor, among others, in the release of these bioactive compounds from the matrix.

Circular economy approaches present several possible applications to improve the sustainability. One of the most advantageous for the food industry is the reintroduction of by-products as ingredients into the food chain. The many advantages of these applications include environmental, social, and nutritional benefits for consumers, as the new ingredients can be used in a wide variety of industrial sectors to enrich foods and improve their technological and nutritional properties. Thus, fortification with ingredients rich in fibre and bioactive compounds may improve the preservation capacity of foods, increasing their shelf life due to the antioxidant effect and water-retention capacity provided by fibre, and improve health, as both compounds help to prevent several non-communicable diseases. However, the fact remains that common ingredients, such as commercial wholemeal flours, which are not by-products in themselves, are still used for food fortification. Therefore, it is very important to encourage the development of research projects to obtain new ingredients from by-products and to promote their application in the design of different functional foods.

## Figures and Tables

**Table 1:** Main concepts and definitions regarding by-product generation.

Concept	Definition	Reference
By-product	A substance or object resulting from a production process whose primary purpose is not the production of that substance or object, and which is not considered as waste, for which different conditions must be fulfilled	[9]
Field by-products	Plant materials that remain after harvesting	[10]
Process by-products	Plant materials discarded during processing because quality standards are not met	[10]
Industrial by-products	By-products generated from different industrial transformation processes of different physical and chemical materials	[10]
Food losses	Losses that take place at production, postharvest, and processing stages in the food supply chain	[2]
Food waste	Losses that take place at the retail and consumption stages at the end of the food supply chain	[2]
Circular economy	An economic model wherein planning, resourcing, procurement, production, and reprocessing are designed and managed, as both process and output, to maximise ecosystem functioning and human well-being	[11]
Bioeconomy	Encompasses the sustainable production of renewable resources from land, fisheries, and aquaculture environments and their conversion into food, feed, fibre, ingredients, bio-based products, and bioenergy, as well as related public goods	[12]

**Table 2:** Dietary fibre (%) and bioactive compounds of fruit and vegetable by-products.

Fruit/Vegetable	TDF *	IDF	SDF	(Poly)phenols	Carotenoids	Glucosinolates	Reference
Pomace							
Apple	51.1	36.5	14.6	10.2 mg GAE/g	-	-	[28]
Redcurrant	58.1	51.1	7.1	20.0 mAU min/g	-	-	[28]
Rowanberry	67.2	59.5	7.7	37.0 mAU min/g	-	-	[28]
Tomato	64.1	58.5	5.6	55.1 mg GAE/g	-	-	[28]
Mango	15	7	8	130 mg GAE/g	-	-	[29]
Peel							
Citrus	67.4	62.5	4.9	1.0 mg GAE/g	-	-	[28]
Mango	69.9	44.2	24.6	0.1 mg GAE/g	5.6 µg β-carotene/g	-	[28]
Plantain	64.3	56.9	7.5	15.2 mg QE/g	-	-	[28]
Tomato	86.2	71.8	14.3	1.6 mg GAE/g	30–40 µg lycopene/g	-	[25]
Seed							
Avocado	3–26	-	-	3–5 mg GAE/g	-	-	[28]
Grapes	8.2	-	-	5 mg/g	-	-	[28]
Papaya	8–9	2.5–3.4	5.2–5.4	34–92 mg GAE/g	-	-	[28]
Stalk							
Broccoli	38.2	35	3.2	1.6 mg GAE/g	-	0.7 mg/g	[30]
Others							
Orange	58.2	46.9	11.3	4.2 mg GAE/g	57.7 µg carotenoids/g	-	[31]
Guava	89.8	86.1	3.7	2.5 mg GAE/g	12.7 µg carotenoids/g	-	[31]
Passion fruit	64.2	44.8	19.4	3.8 mg GAE/g	84.6 µg carotenoids/g	-	[31]
Acerola	48.6	34.4	14.2	19 mg/g	-	-	[32]
Cashew	35	27	8	8 mg/g	-	-	[32]

* Total dietary fibre (TDF); insoluble dietary fibre (IDF); soluble dietary fibre (SDF); gallic acid equivalents (GAEs); mili-absorbance unit (mAU); quercetin equivalents (QEs).
